# Effect of phenolic acids originating from microbes on mitochondria and neutrophils

**DOI:** 10.1186/cc11713

**Published:** 2012-11-14

**Authors:** NV Beloborodova, AY Olenin, NI Fedotcheva, V Shubina, VV Teplova

**Affiliations:** 1 Negovsky Research Institute of General Reanimatology, Russian Academy of Medical Sciences, Moscow, Russia; 2 Lomonosov Moscow State University, Moscow, Russia; 3 Institute of Theoretical and Experimental Biophysics, Russian Academy of Sciences, Pushchino, Moscow Region, Russia

## Background

Several low-molecular-weight phenolic acids are present in the blood of septic patients at high levels [1,2]. The microbial origin of the most of phenylcarboxylic acids in the human body was shown previously [3], but the pathophysiological role of phenolic acids is not clear. It was shown that microbial phenolic acids produce either the antioxidant or the pro-oxidant action on mitochondria depending on the chemical structure [4]. In this work the influence of phenolic acids on reactive oxygen species (ROS) production in mitochondria and neutrophils was investigated.

## Methods

Mitochondria were isolated from the liver of Wistar rats. The difference of electric potentials on the inner mitochondrial membrane (*Δψ*_M_) was determined from the distribution of the lipophilic cation of tetraphenylphosphonium whose concentration in external medium [TPP^+^]_out _was registered using a TPP^+^-selective electrode. The rate of oxygen consumption by mitochondria was determined polarographically. The production of ROS by mitochondria was determined by measuring the chemiluminescence of the Cypridina luciferin. The generation of superoxide anion was induced by 25 μM menadione. The production of ROS by neutrophils was examined by the method of luminol-dependent chemiluminescence. All reagents used were from Sigma (USA). Statistical processing of the data was carried out using the program MS Excel 2003. The differences were considered significant at *P *≤ 0.05.

## Results

ROS production by mitochondria was higher in the presence of cinnamic acid, benzoic acid, 3-phenylpropionic acid and phenylacetic acid than in the presence of menadione alone. The most effective activators of ROS production in mitochondria were those phenolic acids whose effect is mediated via the interaction with thiol (-SH) groups. ROS production by mitochondria was lower in the presence of phenyllactate, *p*-hydroxyphenylacetate or *p*-hydroxyphenyllactate compared with the control. All phenolic acids inhibited ROS formation in neutrophils to different degrees. The inhibition was related to the scavenging of the superoxide anion.

## Conclusion

Microbial metabolites from both the normal gut microbiota and infection sources enter the circulation and can enhance or reduce the inflammatory response. In healthy people phenyllactate and *p*-hydroxyphenyllactate, which decrease ROS production in both in mitochondria and neutrophils, can have the protecting effect on organs and tissues. They can play a role of natural antioxidants. But in septic patients deficit phenolic acids producing the pro-oxidant effects on mitochondria (for example, PPA and PAA) and high levels of other phenols can led to mitochondrial dysfunction and multiorgan failure.
